# Feasibility Study of Soybean Oil-Fortified Foods to Alter Blood Content of Linoleic Acid and Body Weight: A Randomized Double-Masked Placebo-Controlled Crossover Trial

**DOI:** 10.1016/j.tjnut.2025.101288

**Published:** 2025-12-27

**Authors:** Rachel M Cole, Eric Colombo, Austin Angelotti, Genevieve C Sparagna, Rolando E Choriego, Rafael Jimenez-Flores, Andy Ni, Martha A Belury

**Affiliations:** 1 Department of Food Science and Technology, the Ohio State University, Columbus, OH, United States; 2 Department of Nutrition Services, the Ohio State University Wexner Medical Center, the Ohio State University, Columbus, OH, United States; 3 Heart and Vascular Institute, College of Medicine, Penn State University, Hershey, PA, United States; 4 Department of Medicine, Division of Cardiology, University of Colorado Anschutz Medical Center, Aurora, CO, United States; 5 Division of Biostatistics, College of Public Health, the Ohio State University, Columbus, OH, United States

**Keywords:** linoleic acid, fatty acids, soybean oil, dietary oil, cardiolipin

## Abstract

**Background:**

Linoleic acid biomarkers are associated with positive health outcomes including lower risk of diabetes and cardiovascular disease. The effect of consuming linoleate-fortified foods (compared with palm oil-fortified foods) in a randomized controlled, double-masked crossover study to change linoleic acid biomarkers without changing bodyweight is unknown.

**Objectives:**

The aim of this study was to determine the effect of delivering soybean and palm oils through foods on the linoleic acid content of blood fractions and body weight in adults.

**Methods:**

In this crossover pilot study, 4 male and 6 female adults, ages 25–76 y and body mass index from 26 to 41 kg/m^2^, were randomly assigned to consume: 30 g of soybean and palm oil delivered in 3 study foods/d for 4 wk separated by a 2-wk washout period. Bodyweight, fatty acid profile of plasma, erythrocytes, peripheral blood mononuclear cells (PBMC), and dried blood spots, and PBMC cardiolipin were measured before and after each intervention period. Dietary intake was assessed using 24-h recalls. The outcomes were analyzed using the Wilcoxon Signed Rank Test.

**Results:**

After 4 wk of consuming 3 foods/d, plasma linoleic acid decreased during the palm oil intervention (–1.60, *P* = 0.04), whereas it tended to increase in plasma (2.35, *P* = 0.07) and erythrocytes (1.09, *P* = 0.05) during the soybean oil intervention. The percentage of PBMC tetralinoleoyl cardiolipin marginally increased during the soybean oil intervention (2.31, *P* = 0.05) but did not change during the palm oil intervention. There was no difference in energy intake between the 2 interventions (*P* = 0.65) and no change in bodyweight during either intervention (*P* > 0.40).

**Conclusions:**

Foods can be used to deliver 30 g/d of dietary oil for 4 wk to impact linoleic acid biomarkers without incurring body weight changes. These foods are useful for future randomized controlled double-masked clinical trials assessing the impact of dietary oils on energy metabolism.

The study was registered at clinicaltrials.gov as NCT04975763 (https://clinicaltrials.gov/study/NCT04975763).

## Introduction

Linoleic acid (LA) is an essential omega-6 PUFA with a recommended intake of 5%–10% of energy [1]. On average, adults in the United states consume ∼8% of energy from LA [2]. Vegetable oils, salad dressing, and grains are common sources of dietary LA intake [[3], [4], [5]]. However, many oils once rich LA have been replaced by oleic acid-rich varieties [6] suggesting that LA intake in the United states may decline over time [7].

LA biomarkers are associated with reduced risk for cardiovascular disease [8], type 2 diabetes (T2DM) [9], liver fat, visceral adipose tissue [10], and markers of inflammation and insulin resistance [11]. Supplementing the diet with LA can increase lean mass and reduce trunk fat in postmenopausal women with T2DM [12,13]. In a crossover study, when adults consumed a diet rich in LA, insulin sensitivity improved compared with when the same adults consumed a saturated fat-rich diet [14]. In adults with abdominal obesity, a diet rich in LA decreased liver fat and markers of inflammation, both of which were not improved in the group consuming the saturated fat-rich diet. The changes in liver fat were negatively correlated with serum LA [15].

In many tissues, LA is the predominant fatty acid in cardiolipin (CL) [16], a phospholipid located in the inner mitochondrial membrane [17]. CL plays an important role in the inner mitochondria membrane structure [18,19] and in energy production [18,20]. Decreased tetralinoleoyl CL (LA_4_ CL) is often seen in the skeletal muscle of individuals with mitochondrial disease [21], in many tissues including heart and skeletal muscle in patients with Barth syndrome [22,23] and in the heart of adults and youths with cardiomyopathy [24], when compared with healthy controls. Supplementing the diet with oils rich in LA can increase LA_4_ CL in various tissues including rodent livers [25], hearts [[26], [27], [28]], and brown adipose [29], and peripheral blood mononuclear cells (PBMCs) in adults [30]. Supplementing the diet with oils rich in other fatty acids can lead to the incorporation of oleic acid [25,29,31,32] or DHA [25,33,34], instead of LA.

Previous studies have demonstrated that providing LA-rich oils in the diet for 16 wk to postmenopausal women using 8 g oil in supplements [12,13], or 9.3 g oil as loose oil [35], can increase LA biomarkers while not altering weight or BMI. Additionally, when 10 g of LA-rich oil is provided to adults through 1 cookie/d for 2 wk, LA increased in plasma, erythrocyte and PBMC and LA_4_ CL increased in PBMC. However, because of the short duration, weight change was not measured in this study [30]. To understand whether foods delivering LA-rich oil can alter blood LA without altering bodyweight, the objective of this pilot study was to determine changes in biomarkers of LA and bodyweight after 4 wk of consuming 3 foods daily, each made with 10 g of soybean oil (SO) or palm oil (PO). Changes in blood LA and the overall fatty acid profiles including α-linolenic acid, oleic acid, and palmitic acid were assessed. In addition, the ability of participants to consume 3 study foods a day while maintaining successful masking of oil content of study foods was evaluated.

## Methods

### Study design

This 10-wk pilot study was a double-masked randomized placebo-controlled crossover design approved by the Ohio State University Institutional Review Board (2021H0232) and registered on clinicaltrials.gov (NCT04975763). Study data were collected and managed using Research Electronic Data Capture (REDCap) electronic data capture tools hosted at the Ohio State University [36,37]. REDCap is a secure, web-based software platform designed to support data capture for research studies. Recruitment of adults ages 25–80 y took place from August 2021 to December 2022 in the Columbus Ohio area. Participants were initially screened for eligibility through a questionnaire. Inclusion criteria included nonsmoking status, and BMI of 25–55 kg/m^2^. Exclusion criteria included current or previous diagnosis of diabetes, heart, kidney, or liver diseases, some circulatory and autoimmune disease (not including hypotension, hypertension, rheumatoid arthritis, psoriasis, and lupus), current diagnosis of cancer, gastrointestinal disease that influence nutrient digestion and absorption or gastric bypass/bariatric surgery, hypothyroidism or hyperthyroidism diagnosis, unstable medication usage or body weight within the past 3 mo, food allergies or intolerances, dietary restrictions or medication use contraindicated for study foods, current use of weight loss medications, supplements or programs, use of supplements high in LA within the past 4 wk, pregnancy or lactation, inability to draw blood, and alcohol or drug abuse [38]. Recruitment for the study included electronic and flyer postings and ResearchMatch, a national health volunteer registry that was created by several academic institutions and supported by the United States NIH as part of the Clinical Translational Science Award program. ResearchMatch has a large population of volunteers who have consented to be contacted by researchers about health studies for which they may be eligible.

Potential participants reported to the Ohio State University Campus for a screening visit where written informed consent was provided [39]. Participants were assessed for eligibility by measuring height using a wall-mounted stadiometer and weight using a digital scale to determine BMI and by assessing likelihood blood could be collected through venipuncture. A sample of each study food was provided for participants to taste. Eligible participants choosing to continue with the study were scheduled for their first study visit. Product coordinators assigned participants to the study food intervention order (SO then PO; PO then SO) and prepared the study foods for distribution. Participants were randomly assigned to intervention order in blocks of 2 or 4. The randomization scheme was generated by a person outside of the study team.

There were 6 study visits, 1 every 2 wk or 3 during each intervention period (1 at the start, 1 at middle and 1 at the end). Each intervention period lasted 4 wk and was separated by a 2-wk washout period. Participants were asked to submit requested study foods based on their preferences, which were then provided under masked conditions by the study product coordinator. Before each visit, participants were instructed to fast for a minimum of 10 h and to drink water. Blood pressure, pulse, and temperature were measured after 5 min of sitting. Sagittal abdominal diameter (SAD) was measured using a Holtain–Kahn abdominal caliper, and weight was measured using a digital scale. Fasting blood samples were collected at each study visit by venipuncture and a finger stick.

### Study foods

Participants were instructed to consume 3 study foods per day during both 4-wk intervention periods. Study foods included bread rolls, muffins, cookies, brownies, a spread and smoothies. The cookies [30], and smoothies [40] were similar to previous studies using foods to deliver oil. Participants completed a 9-point hedonic scale to evaluate the liking of each baked study food (Supplemental Figure 1). Each study food contained 10 g of either SO or PO for a total of 30 g of the respective oil per day. Nutrient composition (Table 1) was determined using Nutrition Data System for Research software version 2020, developed by the Nutrition Coordinating Center, University of Minnesota. A registered dietitian nutritionist (RDN) instructed participants to maintain their body weight during the study and counseled participants on ways to incorporate the study foods by replacing similar foods in their habitual diet, including providing examples of foods that could be replaced with the study foods and asking each participant for examples of habitual foods that would be easy for them to replace with the study foods. The RDN met with participants every 2 wk to monitor their weight and provide additional counseling for consuming the study food while maintaining body weight as needed. Participants were asked to track their daily study food consumption on a tracking sheet and return any uneaten study foods at the next study visit. Returned tracking sheets were used to determine adherence.

**TABLE 1:** Nutrient information for the study foods.

	Bread roll		Brownie		Cookies		Muffins		Smoothies		Spread	
	SO	PO	SO	PO	SO	PO	SO	PO	SO	PO	SO	PO
Calories (kcal)	275	275	179	179	238	238	205	205	161	161	112	111
Total fat (g)	12	12	12	12	13	13	11	11	10	10	11	11
Saturated fat (g)	2	5	2	6	2	6	2	5	2	5	2	5
16:0 (g)	1	5	1	5	1	5	1	5	1	4	1	5
Polyunsaturated fat (g)	6	1	6	1	6	1	6	1	6	1	6	1
18:2 (g)	6	1	5	1	6	1	5	1	5	1	5	1
18:3 (g)	1	0	1	0	1	0	1	0	1	0	1	0
Monounsaturated fat (g)	3	4	3	4	3	4	3	4	2	4	2	4
18:1 (g)	3	4	3	4	3	4	3	4	2	4	2	4
Total carbohydrates (g)	37	37	19	19	29	29	24	24	17	17	3	3
Dietary fiber (g)	3	3	2	2	3	3	1	1	1	1	0	0
Sugar (g)	4	4	13	13	12	12	12	12	14	14	1	1
Protein (g)	7	7	2	2	4	4	3	3	1	1	1	1

Data expressed as mean for 2 flavors of muffins and 2 flavors of cookies.

Abbreviations: PO, palm oil; SO, soybean oil.

### Fatty acids and CL

Plasma and PBMCs were prepared, and fatty acids were extracted and methylated as we have previously reported [30,41]. In brief, fatty acids were extracted using chloroform and methanol (2:1, v/v) [42], and methylated using hydrochloric acid [43]. Erythrocyte fraction was prepared [30], and fatty acids methylated using 14% boron trifluoride in methanol [[44], [45], [46]], as previously reported [11]. Finger stick whole-blood dried blood spots (DBS) were prepared using DBS card (Whatman 903 Protein Saver Card) treated with butylated hydroxytoluene [47,48]. DBS fatty acid methyl esters were prepared using 14% boron trifluoride in methanol, toluene, and methanol (35:30:35, v/v) [49]. All samples were analyzed by gas chromatography with a flame ionization detector, using a 30-m Omegawax 320 fused silica capillary column (Supelco). Oven temperature started at 175°C and increased at a rate of 3°C/min until reaching 220°C. Flow rate of the carrier gas helium was 30 mL/min [50]. Retention times of samples were compared with standards for fatty acid methyl esters (Matreya, LLC and Nu-Check Prep Inc) [11,30,41]. All identified fatty acids are reported in Supplemental Table 1 for plasma, Supplemental Table 2 for erythrocytes, Supplemental Table 3 for PBMC and Supplemental Table 4 for DBS.

CL fatty acyl molecular species from the isolated PBMC were determined as previously described [51], using electrospray ionization-mass spectrometry coupled to HPLC (LC/MS). In brief, PBMC lipids were isolated using a modified Bligh and Dyer method [52], using 1 μm tetramyristal CL as an internal standard (Avanti Polar Lipids). Samples were analyzed on an API 4000 Qtrap LC/MS (Sciex) using normal phase solutions detailed in Sparagna et al. [51], and full scans. The absolute amounts of the following 72 carbon CL species with mass to charge ratios (*m*/*z*) and LA and oleic acid fatty acyl chains of major species were determined: *m*/*z* 1448 (LA_4_ CL), *m*/*z* 1450 (LA_3_OA_1_ CL), *m*/*z* 1452 (LA_2_OA_2_ CL), and *m*/*z* 1454 (LA_1_OA_3_ CL). Percentages were calculated by dividing by the sum of these species and multiplying by 100, similar to our previous study [30].

### Biochemical analyses

Biochemical markers from blood samples were measured at the start and end of each intervention period according to the manufacturer’s instructions. Fasting serum glucose was measured using the Wako Autokit Glucose kit (Fujifilm Healthcare Americas Corporation), whereas serum insulin was analyzed using MesoScale Diagnostics kit. Plasma total adiponectin, high molecular weight (HMW) adiponectin, and leptin were measured with ELISA kits (Alpco and R&D Systems Inc).

### Diet and activity

Before the start of the first intervention period and between each study visit during both intervention periods, participants were asked to complete three 24-h dietary recalls for a total of 15 dietary recalls and one 24-h activity recall for a total of 5 activity recalls. Recalls were administered on unscheduled days that included both weekdays and weekends. The Automated Self-Administered 24-h Dietary Assessment Tool version 2020 developed by the National Cancer Institute, Bethesda, MD, was used for dietary recalls and the Activities Completed over Time in 24 h [[53], [54], [55]], was used activity recalls. Research staff used the study food tracking sheets that participants returned at each study visit to identify what reported foods in the dietary recalls were study foods and replaced the nutrient composition of those foods with the study food nutrient composition. The Goldberg equations [56], with updated suggested values [57], were used to assess the likelihood that any participant overreported or underreported their dietary intake. Completed dietary recalls at each time point with mean daily kilocalorie intake >1000 were used for analysis. Of those used for analysis, 3 were considered underreporting before intervention period, 2 were considered underreporting while consuming the study foods made with PO and 1 was considered underreporting while consuming the study foods made with SO per the Goldberg equation.

### Blinding index

This was a double-masked study. The success of masking participants and researcher to the oils used in the study food was determined by the Bang Blinding Index [58].

### Statistical analysis

Changes in body weight, fatty acid profiles, and biochemical markers within each 4-wk intervention period were compared with zero using the Wilcoxon Signed Rank Test to assess the effect of each intervention on these outcomes. Differences in changes of these outcomes between interventions were calculated for each participant under the crossover design. These within-participant differences in changes were compared with zero using the Wilcoxon Signed Rank Test to compare the effects of the 2 interventions. To assess the potential of a carryover effect after the 2-wk washout period, a linear mixed effects model was used to determine if there was an interaction between the intervention (SO or PO) and the timing when the intervention was given (before the washout period or after the washout period). If there was an interaction, only data from before the washout period was used to test within-participant differences in changes between interventions using the Wilcoxon Rank-Sum test. Statistical analysis was performed using STATA Versions 18 (StataCorp LLC). *P* values of <0.05 are considered significant.

## Results

Thirteen adults were enrolled in the study, with 12 completing ≥ 1 study visit after the screening visit and were randomly assigned to the order of interventions. Ten participants completed the PO intervention period, and 9 participants completed the SO intervention period (Figure 1). One person discontinued the study before randomization and 1 person discontinued during each intervention period. Of the 10 participants that completed ≥1 study intervention period, 60% were female, the mean age was 47 y and the mean BMI was in the obese category (Table 2).

**FIGURE 1:**
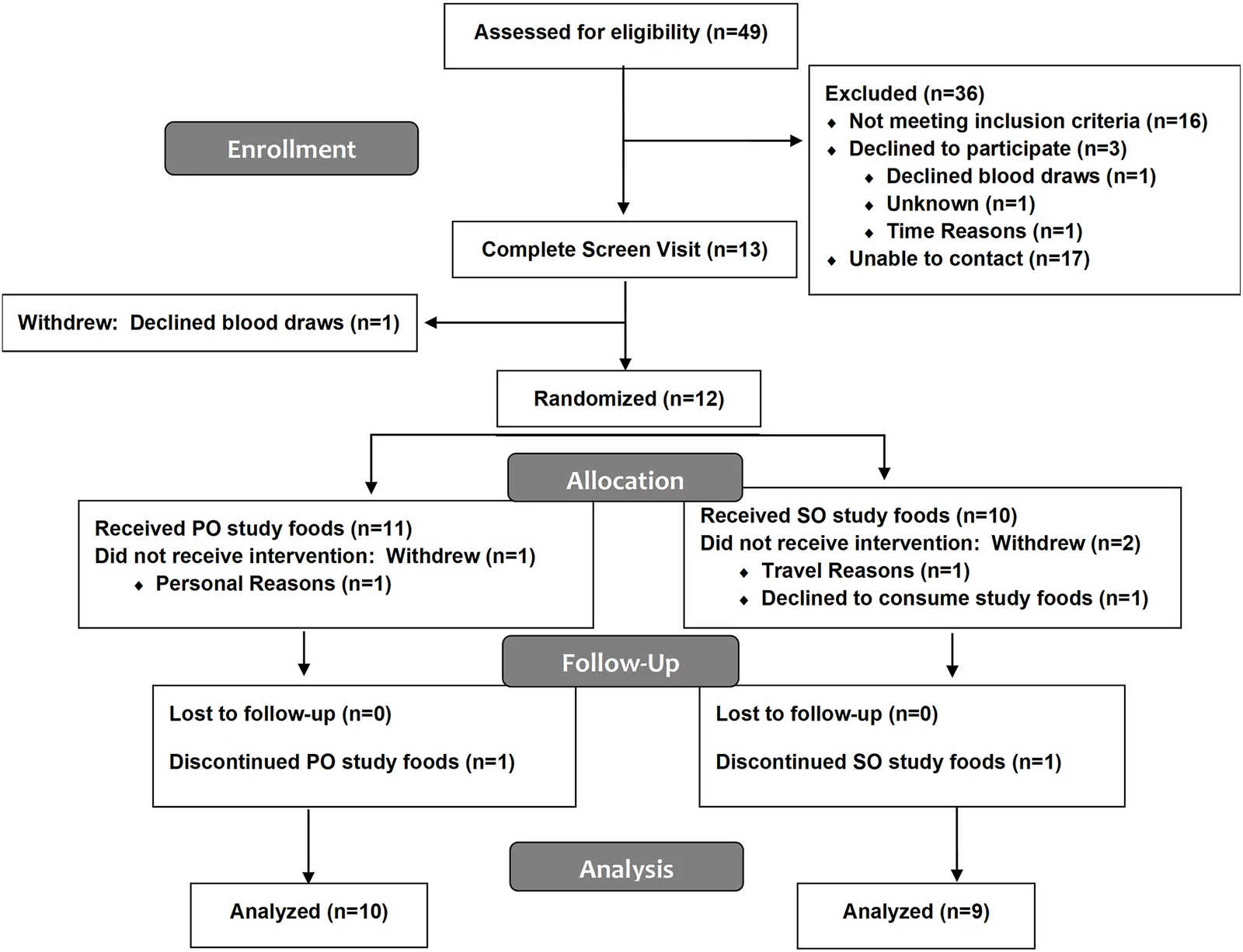
CONSORT flowchart. PO, palm oil; SO, soybean oil.

**TABLE 2:** Participant demographic information (*n* = 10) at screening visit.

Demographic			
Male		4	(40)
Female		6	(60)
Age	47.0	±	17.3
BMI	33.4	±	4.5
Education			
High school diploma/GED		1	(10)
Some college		3	(30)
4-y college degree		1	(10)
Some graduate school		2	(20)
Master's degree		2	(20)
Doctoral degree		1	(10)
Race			
Black		1	(10)
White		8	(80)
Asian		1	(10)
Ethnicity			
Hispanic/Latino		0	(0)
Not Hispanic/Latino		10	(100)

Data expressed as mean ± SD or *n* (%).

Abbreviation: GED, general educational development.

Participants were asked to consume 3 study foods per day for 4 wk during both diet intervention periods. The self-reported mean adherence to consuming 3 study foods per day did not differ between diet periods (*P =* 0.25) at 96.9% during the PO intervention period and 93.4% during the SO period (Figure 2A). The Bang Blinding Index surveyed the participants and researchers about whether they knew which oil was used in the study foods that the participants consumed after each intervention period (Supplemental Tables 5 and 6). For participants, the Bang Blinding Index was –0.1 [95% confidence interval (CI): –0.29, 0.086] for PO and 0.33 (95% CI: 0.025, 0.64) for SO. For the research team, the Bang Blinding Index was 0.2 (95% CI: –0.048, 0.45) for PO and 0.11 (95% CI: –0.094, 0.32). Because the 95% CI exceeded the range of –0.2 to 0.2, that is typically required for successful masking [58], whether masking was successful (or not) for the participants or the research team was inconclusive.

**FIGURE 2:**
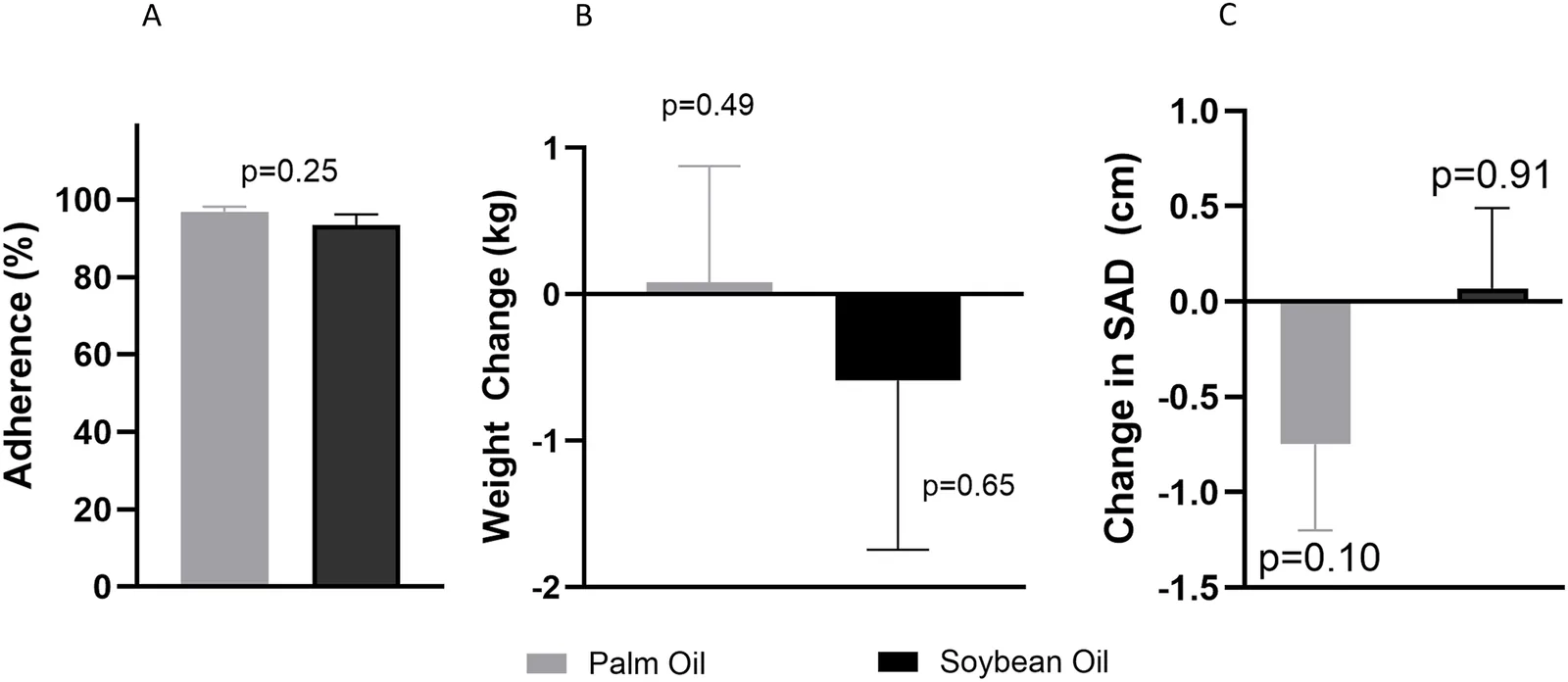
Adherence to study instructions. (A) Self-reported adherence to consuming 3 study foods per day. (B) Change in bodyweight, *P =* 0.36 for within-participant differences in changes between intervention periods; *P* values on graph represent within-participant difference within each intervention period. (C) Change in SAD, *P =* 0.82 for within-participant differences in changes between each intervention period. Data expressed as mean ± SE. SAD, sagittal abdominal diameter.

Participants completed 24-h dietary recalls between visits during each intervention period, and data (excluding kilocalories) were normalized per 1000 kcal (Table 3). Kilocalorie and protein intake were not different between each intervention period (*P =* 0.65, and *P =* 0.43, respectively). Fat intake was borderline lower and carbohydrate intake was borderline higher during the SO intervention period compared with the PO intervention (*P =* 0.10 and *P =* 0.07, respectively). There were differences in saturated fat, monounsaturated fat, and polyunsaturated fat intake adjusted for kilocalories during the 2 intervention periods (*P =* 0.004). This was reflected in the difference in individual fatty acid intake adjusted for kilocalorie intake. Palmitic acid (16:0) and oleic acid (18:1) intakes were higher during the PO intervention period, whereas LA (18:2) and α-linolenic acid (18:3) intakes were higher during the SO intervention period (*P =* 0.004). Compared with baseline diet, during the PO intervention period, saturated fat (*P =* 0.05) and palmitic acid (*P =* 0.002) intake increased and total fat (*P =* 0.08) and oleic acid (*P =* 0.08) intake marginally increased; during the SO intervention period, polyunsaturated fat (*P =* 0.004), LA (*P =* 0.004), and α-linolenic acid (*P =* 0.004) intake increased.

**TABLE 3:** Mean daily intake and mean weekly physical activity during intervention periods.

Diet	Baseline	Intervention period				
		PO	*P* value^1^	SO	*P* value^1^	*P* value^2^
Kilocalories	2204 ± 677	2217 ± 675	0.846	2203 ± 543	1.00	0.652
Protein (g/1000 kcal)	40.1 ± 16.8	36.8 ± 6.8	0.846	33.5 ± 4.7	0.301	0.426
Total carbohydrates (g/1000 kcal)	110.4 ± 17.6	103.2 ± 10.3	0.322	110.5 ± 8.1	0.910	0.074
Total fat (g/1000kcal)	44.2 ± 7.0	49.6 ± 6.0	0.084	47.4 ± 6.3	0.250	0.098
Total saturated fat (g/1000 kcal)	14.9 ± 4.4	19.3 ± 3.2	0.049	13.1 ± 2.3	0.301	0.004
16:0 (g/1000 kcal)	7.6 ± 1.2	13.1 ± 2.2	0.002	7.4 ± 1.1	0.652	0.004
Total monounsaturated fat g/1000 kcal)	15.6 ± 2.3	17.9 ± 2.5	0.131	14.7 ± 2.3	0.496	0.004
18:1 (g/1000 kcal)	14.6 ± 2.1	17.0 ± 2.4	0.084	13.8 ± 2.3	0.652	0.004
Total polyunsaturated fat (g/1000 kcal)	9.9 ± 2.2	8.7 ± 2.2	0.131	16.0 ± 2.6	0.004	0.004
18:2 (g/1000 kcal)	8.7 ± 2.0	7.8 ± 2.0	0.193	14.2 ± 2.4	0.004	0.004
18:3 (g/1000 kcal)	0.9 ± 0.3	0.7 ± 0.3	0.193	1.6 ± 0.3	0.004	0.004
Physical activity						
METs	39.8 ± 7.9	37.0 ± 5.8	0.160	39.4 ± 6.8	0.426	0.129

Data expressed as mean ± SD.

Abbreviations: MET, metabolic equivalent of task; PO, palm oil; SO, soybean oil.

1 *P* value for difference between baseline and the respective intervention period.

2 *P* value for difference between intervention periods.

Kilocalorie, protein, carbohydrate, and monounsaturated fat intake did not change from baseline during either intervention (Table 3). Physical activity expressed as metabolic equivalent of task did not differ between the 2 intervention periods (*P =* 0.13), nor did it change from baseline during either intervention period. Bodyweight and SAD did not change during the SO (*P =* 0.65 and *P =* 0.91) or PO (*P =* 0.49 and *P =* 0.10) intervention periods, and there was no difference in the within-participant changes for bodyweight (*P =* 0.36) or SAD (*P =* 0.82) (Figure 2B and C).

Plasma, erythrocyte, PBMC, and DBS fatty acids were measured at the beginning and end of each intervention period. After 4 wk of consuming 3 foods per day made with PO, plasma LA decreased (Figure 3A; *P =* 0.04). In contrast, plasma LA marginally increased during the SO intervention period (*P =* 0.07), which was different from the change in plasma LA during the PO intervention (*P =* 0.01). Erythrocyte LA (Figure 3B) did not change during the PO intervention (*P =* 0.77) but tended to increase after consumption of 3 SO foods per day for 4 wk (*P =* 0.05). The change in erythrocyte LA during the 2 intervention periods was borderline different (*P =* 0.08). There was not a significant change of PBMC LA (Figure 3C) during either intervention period (*P =* 0.13 for PO and *P =* 0.38 for SO), and there was no difference in the change between the 2 diet periods (*P =* 0.22). Although there was a difference in the change of DBS linoleic (Figure 3D) between the 2 intervention periods (*P =* 0.04), the was no change in DBS LA within SO or PO treatments (*P =* 0.25 and *P =* 0.19, respectively).

**FIGURE 3:**
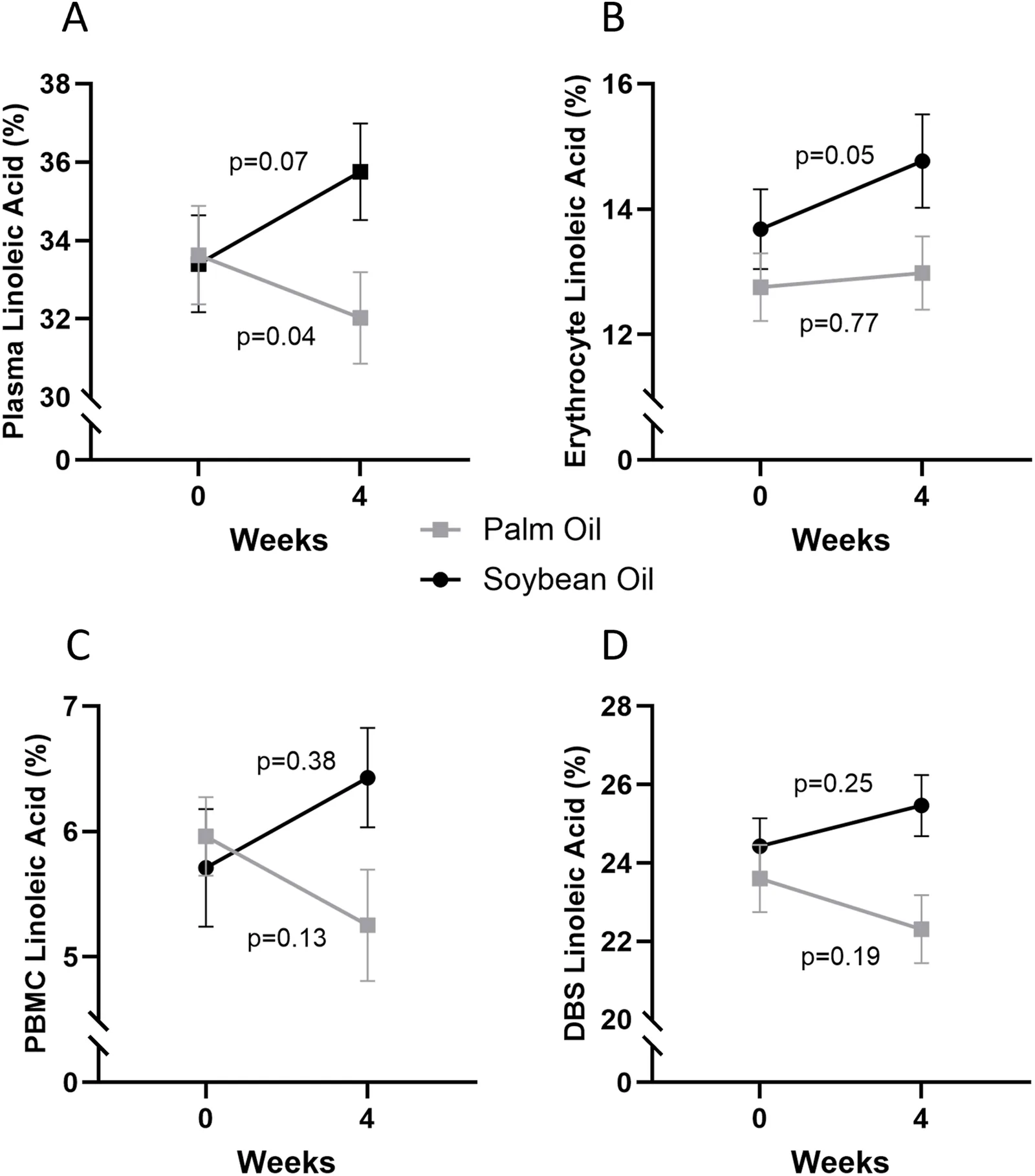
Linoleic acid. (A) Plasma linoleic acid, *P =* 0.01 for within-participant differences in changes between intervention periods. (B) Erythrocyte linoleic acid, *P =* 0.08 for within-participant differences in changes between intervention periods. (C) Peripheral blood mononuclear cell linoleic acid, *P =* 0.22 for within-participant differences in changes between intervention periods. (D) Dried blood spot linoleic acid, *P =* 0.04 for within-participant differences in changes between intervention periods. Data are expressed as mean ± SE; *P* values on graph represent within-participant difference within each intervention period.

There was no change in α-linolenic acid (Figure 4) during the SO or PO intervention periods for plasma (*P =* 0.73 and *P =* 0.23), erythrocytes (*P =* 0.55 and *P =* 1.0) or PBMC (*P =* 0.58 and *P =* 0.49). The differences in changes between the intervention period were also not significant for plasma (*P =* 0.50), erythrocytes (*P =* 0.55), and PBMC (*P =* 0.81). α-linolenic acid decreased in DBS during the PO intervention (*P =* 0.03) but was not changed during the SO intervention (*P =* 0.43). The change in DBS α-LA was not different between each intervention period (*P =* 0.20).

**FIGURE 4:**
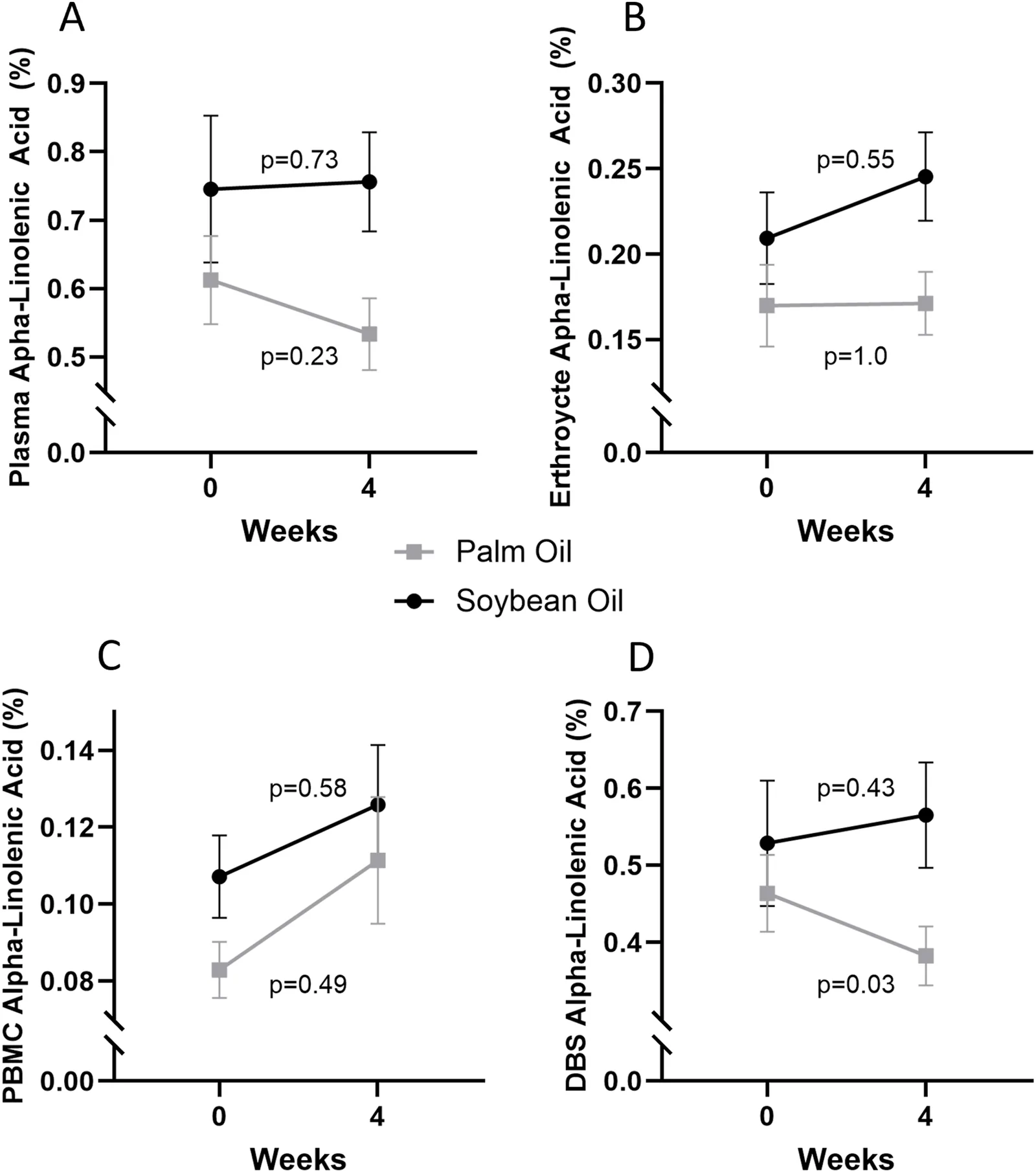
α-linolenic acid. (A) Plasma α-linolenic acid *P =* 0.50 for within-participant differences in changes between intervention periods. (B) Erythrocyte α-linolenic acid *P =* 0.55 for within-participant differences in changes between intervention periods. (C) Peripheral blood mononuclear cell α-linolenic acid *P =* 0.81 for within-participant differences in changes between intervention periods. (D) Dried blood spot α-linolenic acid *P =* 0.20 for within-participant differences in changes between intervention periods. Data are expressed as mean ± SE; *P* values on graph represent within-participant difference within each intervention period.

Erythrocyte and DBS oleic acid (Figure 5B and D) decreased after 4 wk of consuming the SO study foods (*P =* 0.01 and *P =* 0.02, respectively) whereas erythrocyte oleic acid marginally increased during the PO intervention (*P =* 0.08). Additionally, there was a difference in this change of oleic acid between the 2 intervention periods for erythrocytes (*P =* 0.01), and there was a borderline difference for DBS (*P =* 0.07). Oleic acid did not change during either intervention period in both plasma (Figure 5A) and PBMC (Figure 5C), although there was a difference in the change of oleic acid between the 2 intervention periods in plasma (*P =* 0.04), but not PBMC (*P =* 0.81). During the PO intervention period, palmitic acid increased in plasma (Figure 6A), PBMC (Figure 6C), and marginally increased in DBS (Figure 6D) (*P <* 0.01, *P =* 0.03, and *P =* 0.06, respectively), but not in erythrocytes (Figure 6B; *P =* 0.38). These changes were different between the 2 intervention periods for plasma (*P =* 0.03) but not PMBC (*P =* 0.30) or DBS (*P =* 0.13). Palmitic acid in erythrocytes unexpectantly tended to increase in participants from SO foods (*P =* 0.05) but the change during each intervention period was not different (*P =* 0.84).

**FIGURE 5:**
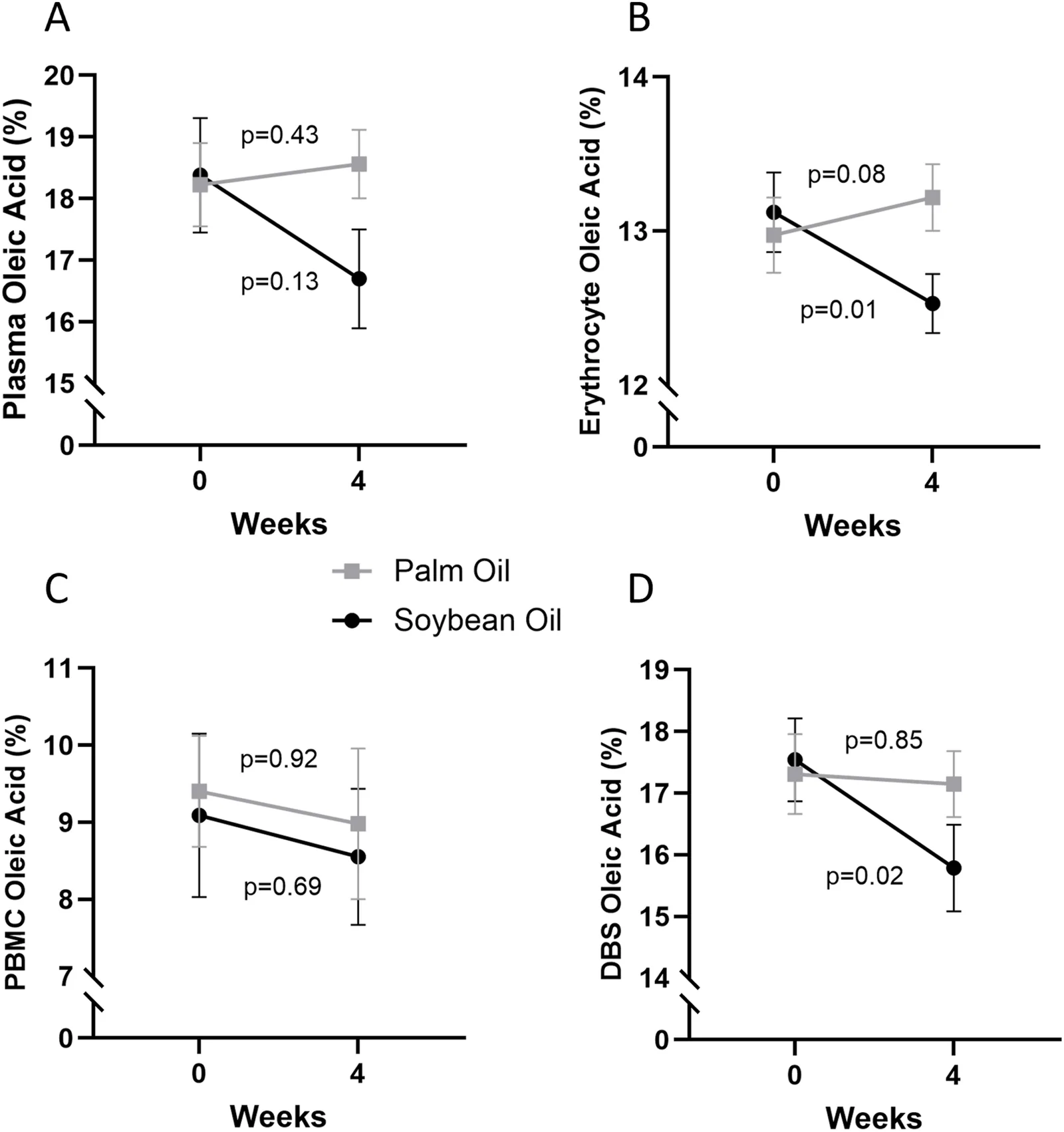
Oleic acid. (A) Plasma oleic acid *P =* 0.04 for within-participant differences in changes between intervention periods. (B) Erythrocyte oleic acid *P =* 0.01 for within-participant differences in changes between intervention periods. (C) Peripheral blood mononuclear cell oleic acid *P =* 0.81 for within-participant differences in changes between intervention periods. (D) Dried blood spot oleic acid *P =* 0.07 for within-participant differences in changes between intervention periods. Data are expressed as mean ± SE; *P* values on graph represent within-participant difference within each intervention period.

**FIGURE 6:**
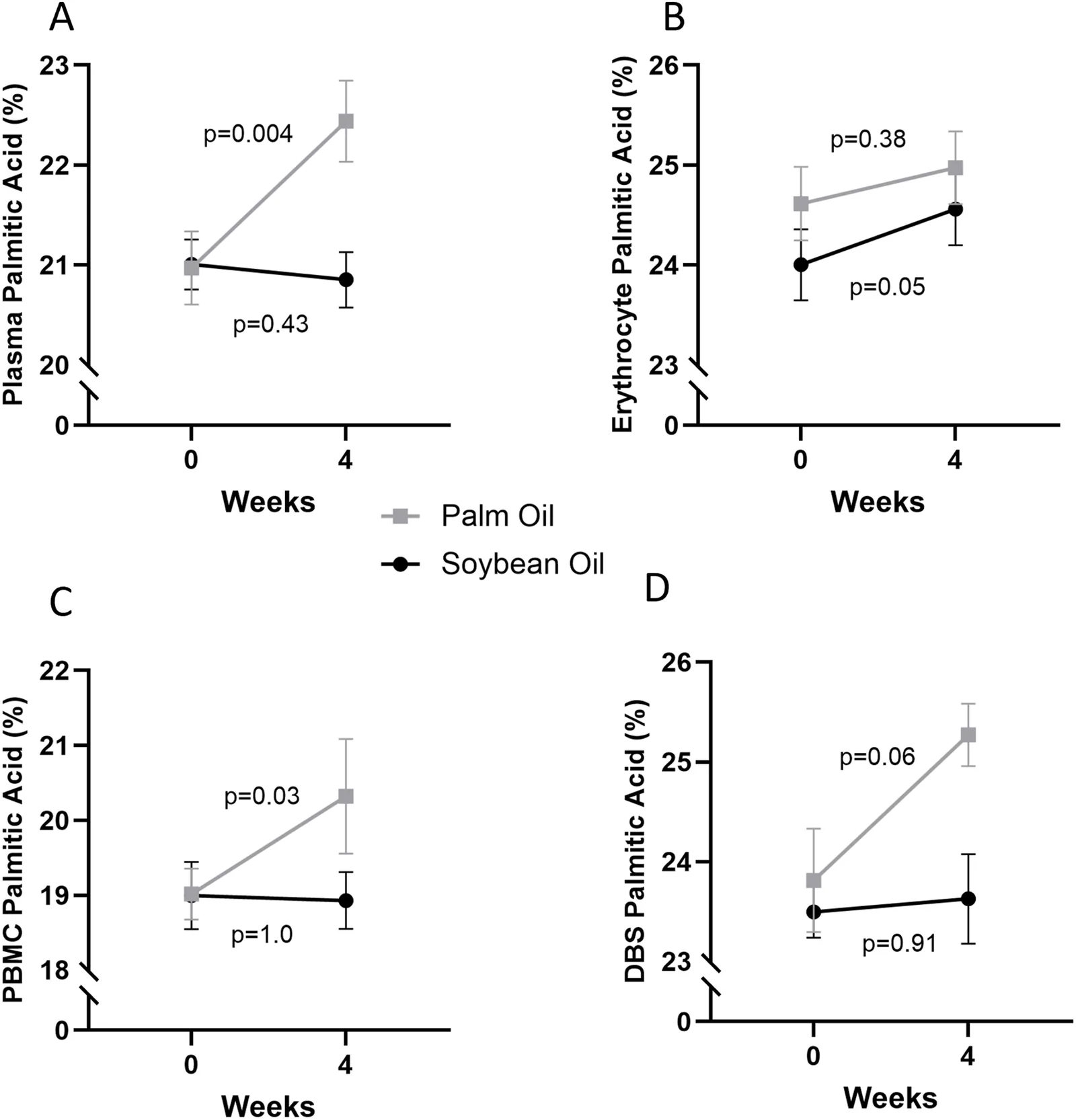
Palmitic acid. (A) Plasma palmitic acid, *P =* 0.03 for within-participant differences in changes between intervention periods. (B) Erythrocyte palmitic acid, *P =* 0.84 for within-participant differences in changes between intervention periods. (C) Peripheral blood mononuclear cell palmitic acid, *P =* 0.30 for within-participant differences in changes between intervention periods. (D) Dried blood spot palmitic acid, *P =* 0.13 for within-participant differences in changes between intervention periods. Data are expressed as mean ± SE; *P* values on graph represent within-participant difference within each intervention period.

CL species were measured in PBMC samples at the start of each intervention period and after 4 wk of consuming 3 study foods per day (Figure 7). The amount adjusted per mg protein of LA_4_ CL did not change during either intervention period (Figure 7A). After 4 wk of consuming 3 study foods per day, the percent of LA_4_ CL (Figure 7B) tended to increase in the SO intervention period (*P =* 0.05) but did not change during the PO intervention (*P =* 0.32). The within-participant changes were not different across the 2 intervention periods (*P =* 0.11). The difference in change of LA_3_OA_1_ CL (Figure 7C) between the 2 intervention periods was marginally different (*P =* 0.05) although the change within either intervention period was not statistically different (*P =* 0.43 for PO and *P =* 0.31 for SO). During the PO intervention, LA_1_OA_3_ CL (Figure 7E) marginally increased (*P =* 0.08) but did not change during SO intervention (*P =* 0.74). The change LA_1_OA_3_ CL was not different between the 2 intervention periods (*P =* 0.38). There was no change in LA_2_OA_2_ CL (Figure 7D) during either intervention period.

**FIGURE 7:**
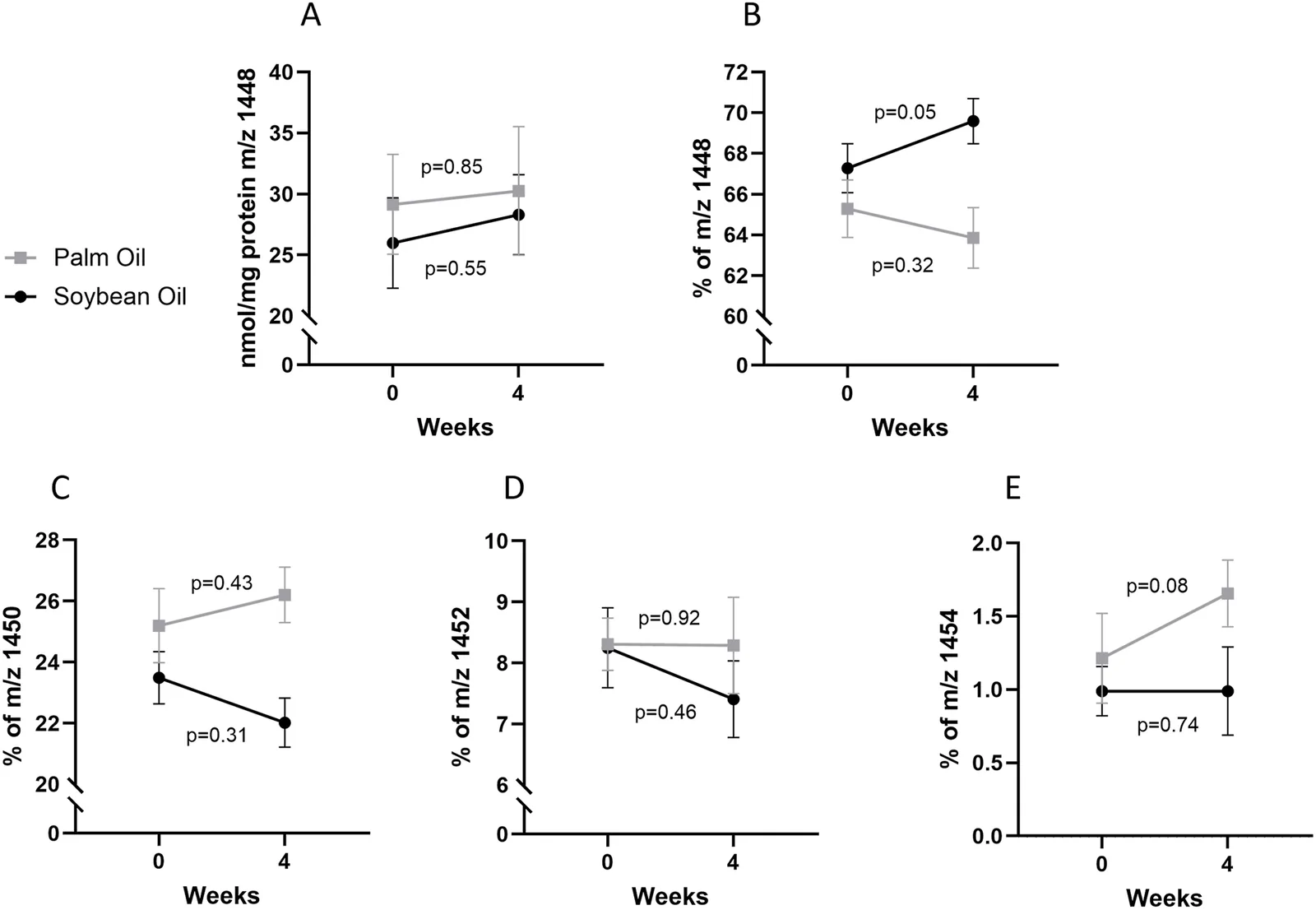
Peripheral blood mononuclear cell cardiolipin species: (A) LA_4_ CL (m/z 1448) cardiolipin concentration, *P =* 0.46 for within-participant differences in changes between intervention periods. (B) LA_4_ CL (m/z 1448) percent *P =* 0.11 for within-participant differences in changes between intervention periods. (C) LA_3_OA_1_ CL (*m*/*z* 1450) percent, *P =* 0.05 for within-participant differences in changes between intervention periods. (D) LA_2_OA_2_ CL (*m*/*z* 1452) *P =* 0.64 for within-participant differences in changes between intervention periods. (E) LA_1_OA_3_ CL (*m*/*z* 1454) percent, *P =* 0.38 for within-participant differences in changes between intervention periods. Percentages were calculated by dividing by the sum of the 4 species and multiplying by 100. Data are expressed as mean ± SE; *P* values on graph represent within-participant difference within each intervention period. LA_4_ CL, tetralinoleoyl cardiolipin.

Markers of glycemia (Supplementary Figure 2) and adipokines (Supplementary Figure 3) were measured in serum and plasma before and after 4 wk of consuming 3 study foods per day. Fasting glucose levels increased during the SO intervention period (p = 0.03) although this change was not different than the PO intervention (p = 0.88) period where no change was observed (p = 0.95). There was no change in insulin or HOMA-IR during either intervention period and no difference in the change between the 2 intervention periods. There was a difference in the change of total adiponectin concentrations between the 2 diet intervention groups (*P =* 0.05), but no significant change during each intervention period. There was no change in either intervention period nor a difference in change between the 2 intervention periods for HMW adiponectin or leptin.

## Discussion

In this randomized double-masked, crossover pilot study, 4 wk of fortifying the diet with 30 g of SO (compared with PO) through consuming 3 foods daily tended to increase LA and LA_4_ CL in the blood without altering bodyweight. Participants reported over 90% adherence to consuming 3 study foods per day for 4 wk without altering mean daily caloric intake. Additionally, the changes in fat composition of their diets during each intervention period reflected the oils used in the study foods consumed. This, combined with no changes in protein intake and marginal changes to total fat intake (PO compared with SO and baseline) and carbohydrate intake (SO compared with PO), supports the usage of these foods to deliver dietary oils in future clinical trials. Future studies should also evaluate the success of masking study foods as carriers of soybean or palm oils, which was inconclusive using the Band Blinding index in this pilot study, possibly due to the small sample size. The development of foods that are able to increase LA in the blood could prove to have cardiometabolic benefits given that biomarkers of LA are inversely related to abdominal obesity [59], waist circumference [60], BMI, LDL-cholesterol, fasting glucose [61], HOMA-IR [62], total mortality [63], risk of stroke [64], and risk of T2DM [9,65].

Both plasma and erythrocyte LA marginally increased after 4 wk of consuming 30 g of LA-rich SO. On the other hand, consuming 30 g of PO daily decreased plasma LA, but did not alter LA in erythrocytes, PBMC or DBS. We previously reported that adults consuming foods fortified with 10 g LA-rich oil for 2 wk increased LA in erythrocytes, PBMC and plasma [30]. The lack of changes of LA in PBMC and DBS observed in the present study may be related to the small sample size or the larger mean BMI in this study compared with our previous study conducted in adults with a lower mean BMI [30]. Accumulation of other fatty acids like DHA and EPA after supplementation has been shown to be influenced by bodyweight [66]; thus, it is possible that larger BMI, and therefore larger blood volume, may influence blood biomarkers sensitivity for LA.

In a previous study, we reported that consumption of a study food fortified with 10 g of a LA-rich oil (compared with comparative oil) every day for 2 wk increased the proportion of LA_4_ CL in PBMCs while decreasing LA_3_OA_1_ CL [30]. Similarly, in this study, consumption of 30 g of SO per day for 4 wk tended to increase LA_4_ CL, whereas consumption of 30 g of PO per day marginally increased LA_1_OA_3_ CL. Several animal studies have demonstrated that diets fortified with LA increase LA_4_ CL, which was also linked with a positive effect on mitochondria function [25,27,29] and some markers of metabolism [29]. In an animal model of heart failure, a diet made with high-LA safflower oil increased cardiac LA_4_ CL and mitochondria function compared with a lard diet [27]. A diet rich in LA compared with a lard diet in mice increased LA_4_ CL and mitochondrial respiration in brown adipose tissue [29], and liver tissue [25]. This increase in brown adipose LA_4_ CL was also accompanied by a decrease in fat mass and improved insulin sensitive [29]. The potential beneficial metabolic effects of increasing LA_4_ CL in human blood and tissue through LA supplementation warrant further study.

During the SO intervention, α-linolenic acid was unaltered in plasma, erythrocytes, PMBC and DBS samples; in contrast, α-linolenic acid decreased in DBS during the PO intervention. In previous intervention studies using SO, the alteration of α-linolenic acid biomarkers has been mixed [67,68]. A study in breast cancer survivors found that consuming 6 g of SO per day for 6 wk did not increase serum α-linolenic acid [67], whereas children of families given a month supply of SO for usage in cooking showed an increase in whole-blood α-linolenic acid [68].

During the PO intervention, oleic acid was unaltered in the blood, whereas palmitic acid increased in plasma and PBMC. During the SO intervention period, oleic acid decreased in erythrocytes and DBS, similar to our previous study showing a decrease in plasma and erythrocyte oleic acid with consumption of foods made with LA-rich oil [30]. Palmitic acid in all blood fractions remained unchanged during the SO intervention period, although in erythrocytes, there was a borderline, unexpected increase. This could be related to the marginal increase in carbohydrate intake during the SO intervention period. In adults with metabolic syndrome, plasma palmitic acid increased when carbohydrate intake was increased [69]. However, a larger study is needed to further investigate the interaction effects of SO and carbohydrate intake on palmitic acid in the blood. Higher palmitic acid in the blood is associated with higher adiposity, markers of inflammation, and insulin resistance as well as risk of diabetes [70]. Biomarkers of oleic acid, on the other hand, have not been found to be associated with either an increased or decreased risk of diabetes [70], altered body composition and/or markers of insulin resistance [11].

Fasting insulin and HOMA-IR were unaltered during the SO or PO intervention periods. Fasting glucose did not change during the PO intervention period, but unexpectantly increased during the SO period. However, the mean fasting glucose at the end of the SO intervention period was still below 100 mg/dl. Results from previous studies using LA-rich oils in different populations are conflicting [12,13,30,35]. In women with metabolic syndrome, a temporary increase in fasting glucose concentrations was demonstrated with consumption of a LA-rich oil that was resolved to baseline levels by week 16 [35]; in women with T2DM, fasting glucose concentrations decreased after 16 wk of supplementation with a LA-rich oil [12,13]; in healthy adults, there was no change in fasting glucose concentrations after 2 wk of consuming a cookie daily made with an LA-rich oil [30]. Additional research in larger and diverse study populations is needed to better understand the effects of LA-rich oil on fasting blood glucose concentrations in adults with obesity but not diabetes.

There were no effects of either SO or PO-fortified foods on adipokines measured in this study. These findings align with findings in a short-term larger study with healthy individuals where consumption of 10 g of an LA-rich oil daily for 2 wk did not alter leptin or total or HMW adiponectin [30]; in contrast, postmenopausal women with metabolic syndrome had an increase in both total and HWM adiponectin after 4 wk of consuming 9.3 g of an LA-rich oil daily [35]. In women with T2DM, adiponectin increased after 12 wk, but not sooner, after daily consumption of 8 g of a high-LA oil [12,13]. It is unknown whether LA-rich oils may increase adiponectin in a longer-term clinical intervention trial.

Strengths of the current study include the crossover design allowing each participant to serve as their own control and the delivery of the oil through foods, which allows for the consumption of larger amounts of oil with less frequency than supplements and a reduced burden on participants to measure or incorporate loose oil into their diets. There are, however, several limitations including the small number of participants, which were from a homogenous group not representative of a diverse group of adults. Finally, the short duration of the intervention periods and washout period justify the need for a larger study with longer intervention and washout durations.

In summary, the findings from this randomized crossover pilot study suggest that using foods to deliver 30 g of SO will alter blood and mitochondrial LA in adults without changing body weight. These foods may be used in future studies to understand if LA-rich oils can have a role in altering the mitochondrial lipidome and respiration, body composition, and energy metabolism particularly in populations that may have impairments in metabolism including sarcopenia, metabolic dysfunction-associated steatotic liver disease.

## Author contributions

The authors’ responsibilities were as follows – MAB, RMC: designed the study; AN, MAB, RMC: wrote the paper, AN, RMC: analyzed data and performed statistical analysis; AA, EC, GCS, REC, RJ-F, RMC: conducted the research; MAB: has primary responsibility for final content; and all authors: read and approved the final manuscript.

## Data availability

Data described in the manuscript, code book, and analytic code will be made available on request pending application and approval.

## Funding

This research was funded by the United Soybean Board (2411-108-0101), Soy Nutrition Institute Global, the Ohio Agriculture 335 Research and Development Center and the National Center for Advancing Translational Sciences, Grant 336 UM1TR004548. Soybean oil was donated by Cargill, Incorporated. The sponsors had no role in the design or conduct of the study, the data analysis, or the decision to publish.

## Conflict of interest

MAB reports financial support was provided by United Soybean Board. MAB reports equipment, drugs, or supplies was provided by Cargill Inc. MAB reports a relationship with United Soybean Board that includes: speaking and lecture fees. All other authors report no conflicts of interest.
