# Supplementary material for: Feasibility Study of Soybean Oil-Fortified Foods to Alter Blood Content of Linoleic Acid and Body Weight: A Randomized Double-Masked Placebo-Controlled Crossover Trial
Source: J Nutr. 2025 Dec 27;156(2):101288. doi: 10.1016/j.tjnut.2025.101288 (PMC13639626; doi:10.1016/j.tjnut.2025.101288)
Supplement: Supplementary file 1 [file mmc1.docx]

**Feasibility Study of Soybean Oil Fortified Foods to Alter Blood Content of Linoleic Acid and Body weight: A Randomized Double-Masked Placebo Controlled Crossover Trial** Rachel M Cole PhD, RDN^1^

**Supplemental Table 1: Plasma Fatty Acid Profile**

|  | Palm Oil Diet Period | | | | Soybean Oil Diet Period | | | |  |
| --- | --- | --- | --- | --- | --- | --- | --- | --- | --- |
|  | Week 0 | Week 4 | Change | Within diet  p-value | Week 0 | Week 4 | Change | Within diet  p-value | Between diet p-value |
| C12:0 | 0.09 (0.1) | 0.06 (0.04) | -0.03 (0.1) | 0.56 | 0.05 (0.02) | 0.09 (0.1) | 0.03 (0.1) | 0.50 | 0.57 |
| C14:0 | 1.21 (0.3) | 1.15 (0.3) | -0.07 (0.3) | 0.49 | 1.25 (0.3) | 1.11 (0.3) | -0.14 (0.4) | 0.13 | 0.65 |
| C16:0 | 20.97 (1.2) | 22.44 (1.3) | 1.47 (1.1) | 0.004 | 21.01 (0.8) | 20.85 (0.8) | -0.15 (1.2) | 0.43 | 0.03 |
| C16:1n7 | 1.46 (0.5) | 1.49 (0.4) | 0.03 (0.2) | 0.70 | 1.38 (0.4) | 1.28 (0.5) | -0.09 (0.3) | 0.36 | 0.43 |
| C18:0 | 6.90 (0.5) | 6.99 (1.2) | 0.10 (0.9) | 0.70 | 6.98 (0.7) | 7.01 (0.4) | 0.03 (0.4) | 0.73 | 1.0 |
| C18:1n9 | 18.22 (2.1) | 18.56 (1.8) | 0.33 (1.5) | 0.43 | 18.38 (2.8) | 16.70 (2.4) | -1.68 (2.7) | 0.13 | 0.04 |
| C18:1n7 | 1.43 (0.2) | 1.34 (0.2) | -0.09 (0.1) | 0.06 | 1.46 (0.13) | 1.37 (0.1) | -0.08 (0.1) | 0.07 | 0.43 |
| C18:2n6 | 33.62 (4.0) | 32.02 (3.7) | -1.60 (1.9) | 0.04 | 33.40 (3.7) | 35.75 (3.7) | 2.35 (3.0) | 0.07 | 0.01 |
| C18:3n6 | 0.47 (0.2) | 0.47 (0.2) | -0.001 (0.2) | 1.0 | 0.42 (0.1) | 0.45 (0.2) | 0.03 (0.2) | 0.73 | 0.91 |
| C18:3n3 | 0.61 (0.2) | 0.53 (0.2) | -0.08 (0.2) | 0.23 | 0.75 (0.3) | 0.76 (0.2) | 0.01 (0.2) | 0.73 | 0.50 |
| C20:0 | 0.33 (0.04) | 0.33 (0.04) | 0.001 (0.04) | 0.77 | 0.40 (0.1) | 0.34 (0.1) | -0.06 (0.2) | 0.36 | 0.57 |
| C20:1n9 | 0.11 (0.03) | 0.11 (0.01) | 0.001 (0.02) | 0.92 | 0.13 (0.04) | 0.10 (0.02) | -0.03 (0.04) | 0.03 | 0.07 |
| C20:2n6 | 0.18 (0.04) | 0.17 (0.04) | -0.01 (0.02) | 0.08 | 0.17 (0.04) | 0.19 (0.05) | 0.01 (0.04) | 0.16 | 0.07 |
| C20:3n6 | 1.41 (0.4) | 1.47 (0.4) | 0.05 (0.3) | 0.92 | 1.36 (0.3) | 1.35 (0.3) | -0.01 (0.2) | 0.57 | 0.73 |
| C20:4n6 | 8.13 (1.4) | 7.87 (1.8) | -0.26 (0.7) | 0.43 | 7.97 (1.9) | 7.93 (2.0) | -0.03 (1.1) | 1.0 | 0.50 |
| C20:5n3 | 0.44 (0.3) | 0.48 (0.30 | 0.04 (0.3) | 0.85 | 0.37 (0.1) | 0.42 (0.2) | 0.05 (0.2) | 0.73 | 0.82 |
| C22:0 | 0.84 (0.2) | 0.83 (0.2) | -0.01 (0.1) | 0.85 | 0.93 (0.2) | 0.81 (0.1) | -0.12 (0.2) | 0.25 | 0.36 |
| C22:4n6 | 0.22 (0.1) | 0.20 (0.1) | -0.01 (0.05) | 0.56 | 0.21 (0.1) | 0.22 (0.1) | 0.02 (0.04) | 0.30 | 0.2 |
| C22:5n6 | 0.19 (0.1) | 0.20 (0.1) | 0.01 (0.1) | 0.70 | 0.19 (0.1) | 0.18 (0.1) | -0.01 (0.1) | 0.73 | 0.65 |
| C22:5n3 | 0.36 (0.1) | 0.37 (0.1) | 0.01 (0.1) | 0.70 | 0.34 (0.1) | 0.39 (0.1) | 0.05 (0.1) | 0.30 | 0.57 |
| C24:0 | 0.78 (0.1) | 0.79 (0.1) | 0.01 (0.1) | 0.77 | 0.90 (0.2) | 0.80 (0.1) | -0.11 (0.2) | 0.36 | 0.43 |
| C22:6n3 | 1.26 (0.3) | 1.33 (0.4) | 0.07 (0.2) | 0.38 | 1.24 (0.2) | 1.23 (0.3) | -0.003 (0.2) | 0.73 | 0.50 |
| C24:1n9 | 0.77 (0.2) | 0.81 (0.2) | 0.04 (0.2) | 0.63 | 0.72 (0.1) | 0.67 (0.1) | -0.05 (0.2) | 0.57 | 0.50 |

Data expressed as mean (standard deviation)

**Supplemental Table 2: Erythrocyte Fatty Acid Profile**

|  | Palm Oil Diet Period | | | | Soybean Oil Diet Period | | | |  |
| --- | --- | --- | --- | --- | --- | --- | --- | --- | --- |
|  | Week 0 | Week 4 | Change | Within diet  p-value | Week 0 | Week 4 | Change | Within diet  p-value | Between diet p-value |
| C16:0 | 24.62 (1.2) | 24.97 (1.2) | 0.36 (1.4) | 0.38 | 24.00 (1.0) | 24.56 (1.0) | 0.55 (0.6) | 0.05 | 0.84 |
| C16:1n7 | 0.31 (0.1) | 0.33 (0.1) | 0.02 (0.1) | 0.43 | 0.30 (0.1) | 0.31 (0.1) | 0.01 (0.1) | 0.74 | 0.95 |
| C18:0 | 20.46 (0.7) | 19.70 (0.7) | -0.76 (0.8) | 0.02 | 19.39 (0.5) | 19.80 (0.9) | 0.41 (0.6) | 0.20 | 0.02 |
| C18:1n9 | 12.97 (0.8) | 13.22 (0.7) | 0.24 (0.4) | 0.08 | 13.12 (0.7) | 12.53 (0.5) | -0.59 (0.4) | 0.01 | 0.01 |
| C18:1n7 | 1.23 (0.1) | 1.18 (0.1) | -0.05 (0.1) | 0.08 | 1.26 (0.1) | 1.20 (0.1) | -0.06 (0.1) | 0.05 | 0.74 |
| C18:2n6 | 12.76 (1.7) | 12.98 (1.8) | 0.22 (1.1) | 0.77 | 13.68 (1.8) | 14.77 (2.1) | 1.09 (1.4) | 0.05 | 0.08 |
| C18:3n3 | 0.17 (0.1) | 0.17 (0.1) | 0.001 (0.1) | 1.0 | 0.21 (0.1) | 0.24 (0.1) | 0.03 (0.1) | 0.55 | 0.55 |
| C20:1n9 | 0.21 (0.1) | 0.25 (0.03) | 0.05 (0.1) | 0.11 | 0.27 (0.05) | 0.22 (0.1) | -0.05 (0.1) | 0.11 | 0.05 |
| C20:2n6 | 0.20 (0.04) | 0.18 (0.04) | -0.02 (0.03) | 0.08 | 0.21 (0.1) | 0.18 (0.1) | -0.03 (0.1) | 0.64 | 0.95 |
| C20:3n6 | 1.43 (0.3) | 1.48 (0.4) | 0.05 (0.2) | 0.56 | 1.50 (0.3) | 1.48 (0.3) | -0.02 (0.1) | 0.74 | 0.64 |
| C20:4n6 | 15.38 (1.3) | 15.22 (1.0) | -0.16 (1.4) | 0.85 | 15.65 (1.4) | 14.82 (1.3) | -0.83 (0.8) | 0.02 | 0.15 |
| C20:5n3 | 0.29 (0.2) | 0.36 (0.2) | 0.07 (0.2) | 0.63 | 0.34 (0.1) | 0.31 (0.1) | -0.03 (0.1) | 0.20 | 0.15 |
| C22:4n6 | 4.15 (0.8) | 4.07 (0.8) | -0.08 (0.4) | 0.49 | 4.08 (0.8) | 3.87 (0.7) | -0.21 (0.3) | 0.15 | 0.15 |
| C22:5n6 | 0.57 (0.2) | 0.58 (0.2) | 0.02 (0.1) | 0.63 | 0.57 (0.2) | 0.58 (0.2) | 0.002 (0.1) | 0.84 | 0.31 |
| C22:5n3 | 2.19 (0.5) | 2.23 (0.3) | 0.04 (0.4) | 0.38 | 2.09 (0.3) | 2.13 (0.4) | 0.04 (0.2) | 0.95 | 1.0 |
| C22:6n3 | 3.07 (0.6) | 3.08 (0.6) | 0.01 (0.3) | 0.63 | 3.33 (0.7) | 3.01 (0.6) | -0.32 (0.2) | 0.01 | 0.02 |

Data expressed as mean (standard deviation)

**Supplemental Table 3: PBMC Fatty Acid Profile**

|  | Palm Oil Diet Period | | | | Soybean Oil Diet Period | | | |  |
| --- | --- | --- | --- | --- | --- | --- | --- | --- | --- |
|  | Week 0 | Week 4 | Change | Within diet  p-value | Week 0 | Week 4 | Change | Within diet  p-value | Between diet p-value |
| C14:0 | 7.69 (4.2) | 8.79 (7.0) | 1.10 (7.3) | 0.77 | 8.16 (4.5) | 8.63 (4.9) | 0.47 (5.2) | 0.81 | 0.69 |
| C16:0 | 19.01 (1.1) | 20.32 (2.4) | 1.30 (2.0) | 0.03 | 18.99 (1.2) | 18.93 (1.0) | -0.07 (0.9) | 1.0 | 0.30 |
| C16:1n7 | 0.21 (0.04) | 0.19 (0.05) | -0.02 (0.1) | 0.19 | 0.19 (0.1) | 0.17 (0.1) | -0.02 (0.04) | 0.47 | 0.81 |
| C18:0 | 19.89 (0.6) | 19.85 (0.9) | -0.04 (1.0) | 0.77 | 19.65 (1.0) | 20.08 (0.6) | 0.43 (0.5) | 0.11 | 0.47 |
| C18:1n9 | 9.40 (2.3) | 8.98 (3.1) | -0.42 (2.1) | 0.92 | 9.09 (2.8) | 8.55 (2.3) | -0.54 (2.0) | 0.69 | 0.81 |
| C18:1n7 | 1.33 (0.3) | 1.17 (0.3) | -0.17 (0.3) | 0.02 | 1.22 (0.3) | 1.20 (0.2) | -0.01 (0.3) | 0.94 | 0.47 |
| C18:2n6 | 5.96 (1.0) | 5.25 (1.4) | -0.71 (1.5) | 0.13 | 5.71 (1.2) | 6.43 (1.1) | 0.72 (1.6) | 0.38 | 0.22 |
| C18:3n3 | 0.08 (0.02) | 0.11 (0.1) | 0.03 (0.1) | 0.49 | 0.11 (0.03) | 0.13 (0.4) | 0.02 (0.05) | 0.58 | 0.81 |
| C20:0 | 2.10 (0.5) | 2.19 (0.7) | 0.10 (0.6) | 0.85 | 2.58 (1.4) | 2.16 (0.4) | -0.42 (1.3) | 0.69 | 0.81 |
| C20:1n9 | 0.40 (0.1) | 0.38 (0.2) | -0.02 (0.1) | 0.85 | 0.37 (0.1) | 0.35 (0.1) | -0.03 (0.1) | 0.38 | 1.0 |
| C20:2n6 | 0.50 (0.1) | 0.41 (0.1) | -0.09 (0.2) | 0.03 | 0.42 (0.1) | 0.53 (0.1) | 0.10 (0.2) | 0.08 | 0.03 |
| C20:3n6 | 1.20 (0.3) | 1.16 (0.4) | -0.05 (0.3) | 1.0 | 1.19 (0.4) | 1.26 (0.3) | 0.07 (0.3) | 0.58 | 0.47 |
| C20:4n6 | 17.05 (3.2) | 15.88 (5.0) | -1.17 (4.4) | 0.77 | 16.22 (4.4) | 16.21 (3.4) | -0.01 (3.7) | 0.69 | 0.47 |
| C20:5n3 | 2.34 (1.1) | 2.52 (1.3) | 0.17 (1.0) | 1.0 | 2.46 (1.0) | 2.59 (0.9) | 0.14 (0.6) | 0.47 | 0.69 |
| C22:0 | 3.30 (0.6) | 3.40 (0.7) | 0.10 (0.7) | 0.85 | 3.93 (1.3) | 3.34 (0.3) | -0.59 (1.2) | 0.30 | 0.47 |
| C22:4n6 | 2.24 (0.3) | 1.99 (0.6) | -0.25 (0.7) | 0.49 | 2.06 (0.3) | 2.22 (0.4) | 0.17 (0.6) | 0.81 | 0.30 |
| C22:5n6 | 0.49 (0.2) | 0.50 (0.1) | 0.001 (0.1) | 0.85 | 0.51 (0.1) | 0.54 (0.1) | 0.04 (0.2) | 0.81 | 1.0 |
| C22:5n3 | 1.41 (0.3) | 1.33 (0.5) | -0.08 (0.4) | 0.85 | 1.24 (0.2) | 1.36 (0.3) | 0.13 (0.3) | 0.30 | 0.16 |
| C24:0 | 2.87 (0.7) | 3.06 (0.9) | 0.19 (0.8) | 1.0 | 3.51 (1.5) | 2.98 (0.4) | -0.52 (1.3) | 0.58 | 0.22 |
| C22:6n3 | 1.18 (0.4) | 1.22 (0.6) | 0.04 (0.4) | 0.77 | 1.10 (0.4) | 1.13 (0.4) | 0.02 (0.3) | 0.94 | 0.94 |
| C24:1n9 | 1.32 (0.4) | 1.30 (0.5) | -0.02 (0.5) | 0.56 | 1.31 (0.3) | 1.20 (0.3) | -0.10 (0.4) | 0.38 | 0.94 |

Data expressed as mean (standard deviation)

**Supplemental Table 4: DBS Fatty Acid Profile**

|  | Palm Oil Diet Period | | | | Soybean Oil Diet Period | | | |  | |
| --- | --- | --- | --- | --- | --- | --- | --- | --- | --- | --- |
|  | Week 0 | Week 4 | Change | Within diet  p-value | Week 0 | Week 4 | Change | Within diet  p-value | Between diet p-value | |
| C16:0 | 23.81 (1.6) | 25.27 (1.0) | 1.46 (2.1) | 0.06 | 23.50 (0.8) | 23.63 (1.3) | 0.13 (1.3) | 0.91 | 0.13 | |
| C16:1n7 | 1.21 (0.4) | 1.24 (0.3) | 0.03 (0.4) | 0.85 | 1.13 (0.4) | 1.10 (0.4) | -0.03 (0.2) | 0.73 | 0.82 | |
| C18:0 | 14.08 (1.8) | 14.33 (2.4) | 0.25 (3.3) | 0.77 | 13.36 (1.1) | 13.84 (2.0) | 0.48 (1.2) | 0.30 | 1.0 | |
| C18:1n9 | 17.31 (2.0) | 17.15 (1.7) | -0.16 (2.1) | 0.85 | 17.54 (2.0) | 15.78 (2.1) | -1.76 (1.6) | 0.02 | 0.07 | |
| C18:1n7 | 1.43 (0.1) | 1.29 (0.2) | -0.14 (0.1) | 0.01 | 1.43 (0.1) | 1.35 (0.1) | -0.09 (0.1) | 0.01 | 0.13 | |
| C18:2n6 | 23.60 (2.7) | 22.30 (2.8) | -1.29 (2.4) | 0.19 | 24.42 (2.2) | 25.46 (2.3) | 1.04 (2.2) | 0.25 | 0.04 | |
| C18:3n6 | 0.25 (0.1) | 0.23 (0.1) | -0.02 (0.1) | 0.77 | 0.22 (0.1) | 0.24 (0.1) | 0.01 (0.1) | 1.0 | 1.0 | |
| C18:3n3 | 0.46 (0.2) | 0.38 (0.1) | -0.08 (0.1) | 0.03 | 0.53 (0.2) | 0.57 (0.2) | 0.04 (0.2) | 0.43 | 0.20 | |
| C20:0 | 0.26 (0.1) | 0.21 (0.1) | -0.05 (0.1) | 0.38 | 0.23 (0.1) | 0.20 (0.1) | -0.03 (0.1) | 0.43 | 0.73 | |
| C20:1n9 | 0.18 (0.03) | 0.17 (0.02) | -0.01 (0.03) | 0.32 | 0.18 (0.03) | 0.17 (0.1) | -0.01 (0.03) | 0.43 | 0.50 | |
| C20:2n6 | 0.22 (0.1) | 0.17 (0.1) | -0.04 (0.1) | 0.38 | 0.19 (0.1) | 0.21 (0.1) | 0.02 (0.04) | 0.30 | 0.16 | |
| C20:3n6 | 1.32 (0.4) | 1.31 (0.2) | -0.01 (0.2) | 0.85 | 1.46 (0.6) | 1.29 (0.2) | -0.17 (0.5) | 0.43 | 0.13 | |
| C20:4n6 | 10.15 (1.4) | 10.13 (1.9) | -0.02 (1.6) | 0.85 | 10.29 (1.5) | 10.31 (1.7) | 0.02 (1.0) | 1.0 | 0.73 | |
| C20:5n3 | 0.39 (0.2) | 0.36 (0.2) | -0.03 (0.2) | 0.38 | 0.29 (0.1) | 0.34 (0.2) | 0.05 (0.1) | 0.20 | 0.36 | |
| C22:0 | 0.51 (0.2) | 0.55 (0.4) | 0.04 (0.3) | 0.43 | 0.46 (0.2) | 0.53 (0.3) | 0.07 (0.2) | 0.43 | 0.91 |  |
| C22:4n6 | 1.53 (0.3) | 1.61 (0.4) | 0.08 (0.2) | 0.11 | 1.55 (0.4) | 1.64 (0.4) | 0.09 (0.2) | 0.16 | 0.73 |  |
| C22:5n6 | 0.31 (0.1) | 0.31 (0.1) | 0.00 (0.1) | 0.85 | 0.29 (0.2) | 0.33 (0.1) | 0.03 (0.04) | 0.05 | 0.36 |  |
| C22:5n3 | 0.93 (0.2) | 0.98 (0.2) | 0.05 (0.2) | 0.28 | 0.94 (0.2) | 1.01 (0.2) | 0.07 (0.1) | 0.16 | 1.0 |  |
| C24:0 | 0.35 (0.1) | 0.29 (0.1) | -0.06 (0.2) | 0.19 | 0.30 (0.1) | 0.31 (0.2) | 0.02 (0.2) | 1.0 | 0.43 |  |
| C22:6n3 | 1.70 (0.4) | 1.70 (0.6) | 0.005 (0.4) | 0.85 | 1.69 (0.4) | 1.70 (0.5) | 0.01 (0.2) | 1.0 | 0.91 |  |

Data expressed as mean (standard deviation)

**Supplemental Table 5: Bang Blinding Index for Participants.**

| Response | Palm oil group | Soybean oil group |
| --- | --- | --- |
| Guess palm oil | 0 | 0 |
| Guess soybean oil | 1 | 3 |
| Don’t know | 9 | 6 |

Data expressed as number of participants.

**Supplemental Table 6: Bang Blinding Index for Researcher**.

| Response | Palm oil group | Soybean oil group |
| --- | --- | --- |
| Guess palm oil | 2 | 0 |
| Guess soybean oil | 0 | 1 |
| Don’t know | 8 | 8 |

Data expressed as number of participants.

**Supplemental Figure 1: 9-Point Hedonic Scale.** Participants tasted either palm oil foods or soybean oil foods and completed a 9-point hedonic rating of their overall liking of the food**;** 1=dislike extremely, 2=dislike very much, 3=dislike moderately, 4=dislike slightly, 5=neither like nor dislike, 6= like slightly, 7=like moderately, 8=like very much, 9= like extremely. **A** Chocolate chip cookie; **B** Spice Cookie; **C** Blueberry Muffin; **D** Lemon Muffin; **E** Bread; **F** Brownie; Data is expressed as mean ± standard error. Palm oil (PO) and Soybean Oil (SO).

**Supplemental Figure 2:	Markers of Glycemia. A** Fasting Glucose p=0.88 for within-participant differences in changes between intervention periods; **B** Fasting Insulin p=0.63 for within-participant differences in changes between intervention periods; **C** HOMA-IR p=0.13 for within-participant differences in changes between intervention periods. Data is expressed as mean ± standard error; p-values on graph represent within participant difference within each intervention period.

**Supplemental Figure 3:	Adipokine Levels. A** Total Adiponectin p=0.05 for within-participant differences in changes between intervention periods; **B** High Molecular Weight (HMW) Adiponectin p=0.69 for within-participant differences in changes between intervention periods; **C** Leptin p=0.94 for within-participant differences in changes between intervention periods; Data is expressed as mean ± standard error; p-values on graph represent within participant difference within each intervention period.
